# IMPLICATIONS OF MEDICAID NURSING HOME PAYMENT AND COSTS FOR POLICY AND PRACTICE

**DOI:** 10.1093/geroni/igae098.2005

**Published:** 2024-12-31

**Authors:** John Bowblis, Edward Miller, Elizabeth Simpson, Sara Karon, Iara Oliveira, Judith Dey, Marie Squillace, Marc Cohen

**Affiliations:** Miami University, Oxford, Ohio, United States; University of Massachusetts Boston, Boston, Massachusetts, United States; University of Massachusetts Boston, Boston, Massachusetts, United States; RTI International, Research Triangle Park, North Carolina, United States; Office of Behavioral Health, Disability, and Aging Policy (BHDAP), Washington, District of Columbia, United States; US Department of Health and Human Services, Washington, District of Columbia, United States; Office of the Assistant Secretary for Planning and Evaluation, Washington, District of Columbia, United States; University of Massachusetts Boston, Boston, Massachusetts, United States

## Abstract

This study is the most comprehensive analysis to date on the relationship between state Medicaid payment rates and nursing home (NH) reported costs of caring for Medicaid residents. The findings provide insight into how, on average, Medicaid payments compare to reported costs, and how that differs by different types of NHs. It does not address the adequacy of Medicaid payment, whether NHs were operating efficiently or were adequately staffed based on the acuity of their residents; nor does it address the quality of the data from the cost reports. Nonetheless, it does provide important information on state Medicaid payment rates and reported costs that can inform current discussions assessing potential Medicaid payment policy reforms. Findings from this study also can be used to examine the impact of potential changes in Medicaid payment on financial performance and disparities across NHs. Study limitations and directions for further investigation are discussed.

